# Integrating Sister Chromatid Cohesion Establishment to DNA Replication

**DOI:** 10.3390/genes13040625

**Published:** 2022-03-31

**Authors:** Caitlin M. Zuilkoski, Robert V. Skibbens

**Affiliations:** 1 Department of Biological Sciences, Lehigh University, 111 Research Drive, Bethlehem, PA 18015, USA; czuilkos@iu.edu; 2 Department of Biology, Indiana University, 1001 E. Third Street, Bloomington, IN 47401, USA

**Keywords:** Eco1/Ctf7/ESCO2, PCNA, RFC complexes, DNA replication, sister chromatid cohesion

## Abstract

The intersection through which two fundamental processes meet provides a unique vantage point from which to view cellular regulation. On the one hand, DNA replication is at the heart of cell division, generating duplicate chromosomes that allow each daughter cell to inherit a complete copy of the parental genome. Among other factors, the PCNA (proliferating cell nuclear antigen) sliding clamp ensures processive DNA replication during S phase and is essential for cell viability. On the other hand, the process of chromosome segregation during M phase—an act that occurs long after DNA replication—is equally fundamental to a successful cell division. Eco1/Ctf7 ensures that chromosomes faithfully segregate during mitosis, but functions during DNA replication to activate cohesins and thereby establish cohesion between sister chromatids. To achieve this, Eco1 binds PCNA and numerous other DNA replication fork factors that include MCM helicase, Chl1 helicase, and the Rtt101-Mms1-Mms22 E3 ubiquitin ligase. Here, we review the multi-faceted coordination between cohesion establishment and DNA replication. SUMMARY STATEMENT: New findings provide important insights into the mechanisms through which DNA replication and the establishment of sister chromatid cohesion are coupled.

## 1. Introduction: PCNA and RFC Complexes Function in DNA Replication

A crucial process during cell division is the accurate replication of the parental genome. DNA replication requires a full symphony of factors that include MCM helicase, primase, leading and lagging strand DNA polymerases, and Okazaki maturation factors [1,2,3]. Of particular interest is the sliding clamp PCNA (proliferating cell nuclear antigen). PCNA is an essential factor that homo-trimerizes into a closed ring-like structure. As a topologically closed ring, PCNA requires pentameric AAA + ATPase replication factor C complexes (RFCs), which transiently open and load PCNA onto DNA [4,5]. RFCs are five component complexes in which one of several large subunits (Rfc1, Ctf18, or Elg1) bind a core four-subunit assemblage comprising Rfc2-Rfc5. Intriguingly, different RFCs exhibit antagonistic activities. For instance, Rfc1^RFC^ and Ctf18^RFC^ both load PCNA onto DNA [6,7,8]. Once loaded, PCNA acts as a sliding clamp that binds DNA polymerases and ensures progressive DNA replication of the leading strand and back-stitched DNA replication of the lagging strand. In contrast, Elg1^RFC^ dissociates PCNA from DNA, although Rfc1^RFC^ likely exhibits some level of PCNA unloading activity as well [9,10,11]. In addition to binding both leading and lagging strand DNA polymerases, PCNA also provides a landing pad for other factors that include p21, Fen1/Rad27, DNMT1, E3 ligases, Cdc9 ligase, PAF^p15^, Srs2, and Cdt1 [12,13,14,15]. The majority of these PCNA-binding proteins contain a conserved sequence known as the PIP (PCNA-interacting peptide) box, which binds the inter-domain connector loop (IDCL) of PCNA [16]. Non-canonical PIP sequences, and domains outside of the PIP box, however, often augment binding to PCNA [16]. Factors that bind PCNA often do so in a competitive fashion and are regulated, in part, by the post-translational modifications that are present on PCNA [17,18,19,20,21,22]. For example, acetylation of lysine 20 promotes PCNA function in homologous recombination, while ubiquitination of lysine 164 is required for translesion synthesis [23,24,25]. Through these combined associations, PCNA coordinates DNA replication with histone deposition, Okazaki maturation, DNA repair, cell cycle regulation, and other processes that promote genomic integrity.

## 2. Part I—Sister Chromatid Cohesion Is Established by Eco1/Ctf7 and Cohesin

The survival of cell progeny requires that the products of chromosome replication, termed sister chromatids, be identified from the time of replication during S phase until their segregation during mitosis. Sister chromatid identity is mediated through physical tethering, termed cohesion, and is established in a two-step process. First, tethering complexes (termed cohesins, comprising Smc1, Smc3, Mcd1/Scc1, Scc3/Irr1, and Pds5) are loaded onto each nascent sister chromatid during S phase by a deposition heterocomplex termed Scc2, Scc4 (NIPBL and MAU2 in humans) [26,27]. Second, DNA-associated cohesins are converted to a tethering-competent state by a highly conserved acetyltransferase Eco1/Ctf7 [28,29,30,31,32,33,34,35,36]. Humans contain two Eco1 homologs, ESCO1/EFO1 and ESCO2/EFO2, but ESCO2 is largely responsible for cohesion establishment during S phase [37]. Eco1 (and ESCO2) modifies the Smc3 subunit of the cohesin complex during S phase to establish cohesion [32,33,38,39], likely through cohesin dimerization or clustering [33,39,40,41,42,43,44,45]. Cohesin degradation, which defines anaphase onset, enables sister chromatids to segregate away from each other and into the newly forming daughter cells [46]. Intriguingly, Eco1 also acetylates the Mcd1 (RAD21 in humans) subunit of cohesins during G2 and M phases in response to DNA damage. This damage-induced cohesion (DIC) stabilizes sister chromatid proximity and thus promotes strand-invasion and high-fidelity DNA repair through homologous recombination [47]. Eco1 plays an additional role in DNA damage repair during S phase. In response to replicative stress, Eco1 acetylates the inner surface of PCNA, which in turn promotes homologous recombination-based repair [23]. In fission yeast, Eso1 appears as a genetic fusion of Eco1 and Rad30, the latter of which is a translesion/repair DNA polymerase—also known as Polη—that plays an important role during DIC [48,49,50]. Once established during either S or G2/M phase, sister chromatid cohesion is tenacious, resisting significant microtubule spindle forces that produce predominantly poleward-directed forces [51,52,53].

## 3. Part II—Coupling Cohesion Establishment to the DNA Replication Fork through PCNA

**Figure 1:**
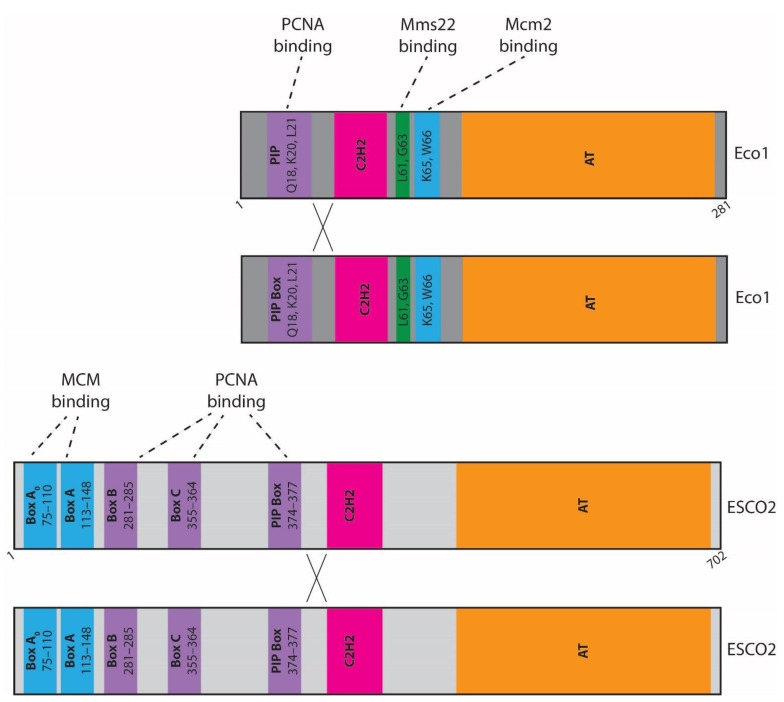
**Eco1/ESCO2 interact with replication factors.** Schematic domain maps of yeast Eco1 (**above**) and *Xenopus* ESCO2 (**below**). C_2_H_2_ indicates the zinc finger motif and AT indicates the acetyltransferase domain. Within Eco1, PCNA binds to amino acids Q18, K20, and L21; Mms22 binds to amino acids L61 and G63; and Mcm2 binds to amino acids K65 and W66. Within ESCO2, MCM binds Box A_0_ and Box A, and PCNA binds to Box B, Box C, and the PIP box. Additional evidence suggests dimerization of Eco1/ESCO2 is important for its function, indicated by the “X”.

Although sister chromatid cohesion is essential for high fidelity chromosome segregation during mitosis, the process of cohesion establishment occurs during S phase. The model that Eco1 promotes cohesin specifically in the context of the DNA replication fork [33,54] has stood the test of time. Founding the field of cohesion establishment were the discoveries that PCNA overexpression rescues *eco1* mutant cell inviability, whereas viable mutations of *pol30* and *eco1* together result in lethality. Together, these findings suggested that PCNA and Eco1 interact directly to couple cohesion establishment to DNA replication [33,54]. Subsequent studies indeed provide compelling evidence that Eco1 binds to PCNA, and a PIP box in Eco1 was identified that directs PCNA binding and promotes cohesion establishment [55,56,57] (Figure 1). Earlier studies, however, portended that other domains likely support Eco1-PCNA association [58]. Careful analyses of human ESCO2 subsequently revealed three additional domains (Box A, B, and C) that reside within the N-terminal region of ESCO2 that supports binding to PCNA [57] (Figure 1). Notably, an N-terminus construct of ESCO2 that contains Box C, but is devoid of Box A, B, and the PIP box, is sufficient to bind PCNA at replication foci and, when tethered to DNA, recruit PCNA to non-replicating chromatin [57].

The role of PCNA in Eco1-dependent sister chromatid cohesion predicts that RFC mutations should exert corresponding effects on cohesion. These predictions are borne out. Deletion of *CTF18*, which encodes the PCNA loader subunit of Ctf18^RFC^, results in reduced levels of chromatin-bound PCNA and increased cohesion defects. Moreover, the combination of non-lethal *ctf18* and *eco1* mutations results in synthetic lethality [7,8,33]. Increasing PCNA levels on chromatin has the opposite effect on *eco1* mutant cells. Deletion of *ELG1*, which encodes the PCNA unloader subunit of Elg1^RFC^ and thus increases levels of chromatin-bound PCNA, rescues *eco1* mutant cell temperature sensitivity, reduces the frequency of cohesion defects, and increases Smc3 acetylation levels [59,60,61,62,63].

Continued studies of replication-coupled cohesion establishment have provided unanticipated insights into DNA replication. Until recently, PCNA association with DNA was thought to be asymmetric, with the leading strand having low levels of bound PCNA compared to the lagging strand. This model is consistent with the notion of long-lived and processive leading-strand DNA replication in which only a single round of PCNA/polymerase recruitment is required. In comparison, the back-stitched DNA replication of the lagging strand requires numerous cycles of PCNA/polymerase recruitment to consecutive primase-initiated sites that resolve during Okazaki fragment maturation [64]. Analyses by eSPAN (enrichment and sequencing of protein-associated nascent DNA), which entails chromatin immunoprecipitations (for instance, PCNA pull-down) followed by BrDU-based immunoprecipitation of single-stranded DNAs, provided strong support for increased levels of PCNA on lagging strands of active replication forks [64,65]. Surprisingly, increased levels of PCNA were observed on leading strands in which fork progression was stalled by exposure to the hydroxyurea [64,65]. Thus, PCNA strand association appears to be dependent on fork activity, and likely PCNA modifications, that arise during replicative stress [65,66]. A more recent eSPAN study, however, found that equal levels of PCNA are present on leading and lagging strands. These latter eSPAN analyses were performed during normal replication fork progression in cells previously synchronized through pheromone treatment [67]. Equivalent PCNA distribution across leading and lagging strands requires independent confirmation, but if true, raises important questions as to the roles that RFC complexes play in normalizing PCNA levels across the replisome. For instance, after confirming that Rfc1^RFC^ is essential for DNA replication, eSPAN analyses mapped Rfc1^RFC^ predominantly to the lagging strand. Thus, the lagging strand population of Rfc1^RFC^, and the PCNA that it loads, appear to function primarily in DNA replication. This finding posed a second question: Does this population of PCNA promote sister chromatid cohesion? Surprisingly, Rfc1^RFC^ appeared not to play a significant role in sister chromatid cohesion. Thus, Rfc1^RFC^-loaded PCNA on the lagging strand is required for DNA replication but may be independent from a role in cohesion establishment [68].

If not through Rfc1^RFC^-loaded PCNA, how does sister chromatid cohesion, which requires PCNA and Eco1 recruitment, get established? Further insights were obtained by the finding that Ctf18^RFC^ maps to the leading strand of the DNA replication fork [67]. Prior findings established that *CTF18* deletion produces a significant adverse effect on sister chromatid cohesion, but not an adverse impact on DNA replication [7,8,33,67,69,70]. Moreover, early studies revealed that Ctf18^RFC^ loads DNA polymerase ε, a leading strand polymerase [71,72]. In combination, these findings suggest that Ctf18^RFC^, and the PCNA that it loads, function primarily in establishing cohesion via strand-biased Eco1 recruitment-independent of significant DNA replication activity performed by Rfc1^RFC^-loaded PCNA. The increased level of leading strand PCNA, via Ctf18^RFC^, is then ultimately normalized to that of lagging strand levels by Elg1^RFC^, which acts to dissociate PCNA from DNA [11,67,73].

## 4. Part III—Additional Replication Factors Couple Cohesion Establishment and the DNA Replication Fork

For many years, PCNA was thought to act as the sole recruiter of Eco1 to the DNA replication fork and thereby couple cohesion establishment to DNA replication [33,55]. Recently, however, several DNA replication fork-associated factors (MCM helicase, Bre1 component of E3 ubiquitin ligase, Rtt101-Mms1-Mms22 E3-ubiquitin ligase complexes, and Chl1 DNA helicase) were identified as recruiting Eco1 and/or promoting Eco1-dependent sister chromatid cohesion [56,61,74,75,76].

### 4.1. MCM Links ESCO2/Eco1 to Both Pre-Replication Complex and Active Replisomes

The PCNA-dependent recruitment model of cohesion establishment predicts that Eco1 is recruited only to actively replicating DNA. Contrary to this model, however, are early findings that Smc3 becomes acetylated by ESCO2/Eco1 prior to DNA replication [56,77]. Given that sister chromatids do not exist prior to S phase, this acetylation reaction appears to be largely inconsequential to cohesion establishment, but is important for highlighting early recruitment of ESCO2/Eco1, independent of PCNA, to origins and pre-replication complexes (pre-RC) [56,77]. Studies in human cell lines confirmed that ESCO2 recruitment to pre-RCs preceded that of PCNA recruitment and instead were correlated with MCM helicase components of pre-RC complexes [78]. The MCM2-7 hexameric helicase complex is an ATPase that moves progressively along DNA, unwinding the double-stranded DNA duplex to form leading and lagging strand templates. MCM helicase, along with the origin recognition complex (ORC), Cdc6, and Cdt1, help comprise the pre-RC complex.

Co-immunoprecipitation and mass spectrometry studies documented that ESCO2 binds all MCM subunits, with yeast two-hybrid identifying specific contacts to Mcm4 and Mcm6 [74,78]. In combination, these findings provide compelling support for a model in which ESCO2 is recruited to origins via MCMs associated with the pre-RCs. One possibility is that ESCO2-MCM helicase interactions are transitory, allowing for ESCO2 to subsequently bind PCNA. This MCM-to-PCNA handoff positions ESCO2 immediately behind the replisome to convert cohesins, newly loaded onto each leading and lagging strand, into a tethering competent state. Notably, MCM helicase association with ESCO2 decreases during S phase [76], in support of this hand-off model. One important attribute of MCM binding is that it protects ESCO2 from degradation by Cullin-RING (CUL4A/B) and anaphase promoting complex (APC) E3 ubiquitin ligases during S phase [78,79]. Once released from the DNA replication fork, and throughout both G2 and M phases, Eco1 is degraded via a stepwise phosphorylation strategy that involves Cdk1, Dbf4-Cdc7 (DDK) kinase, and Mck1/GSK-3 signaling kinase, followed by ubiquitination via SCF ubiquitin ligase [79,80,81,82,83].

**Figure 2:**
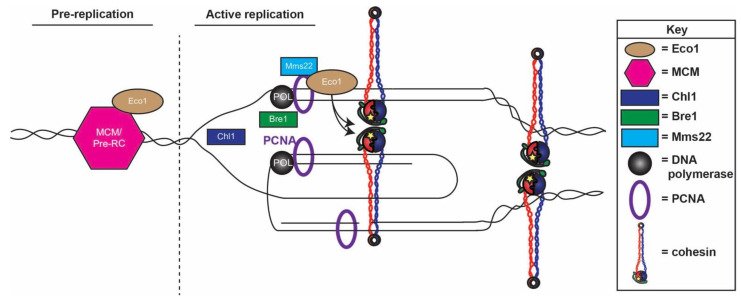
**Eco1 handoff model for cohesion establishment at the DNA replication fork.** The association between Eco1 and the MCM complex at the pre-replication complex positions Eco1 to sites of replication. Once replication is initiated, Eco1 is released from MCMs to associate with Mms22 and PCNA. Augmented by Chl1 and Bre1 E3 ligase at the replication fork, Eco1 acetylates cohesin loaded behind the replication fork to establish sister chromatid cohesion.

How well conserved is ESCO2-MCM binding and how is the hand-off between ESCO2 and MCM helicase and PCNA regulated? As described above, numerous motifs (Box B, C, and the PIP box) within the N-terminus of ESCO2 promote PCNA binding and recruitment to the DNA replication fork [57] (Figure 1). Further analyses, however, revealed that Box A (amino acids 113–148) is essential for ESCO2 binding to Mcm4 [57]. In addition, amino acids 75–110 were subsequently identified as promoting the binding of ESCO2 to Mcm4, Mcm5, Mcm6, and Mcm7 [74,78]. Herein, we refer to this newly identified region as Box A_0_, since it is N-terminal to Box A. The N-terminal portion of ESCO2 that contains MCM-binding Boxes A_0_ and A (as well as Boxes B and C that promote binding to PCNA), however, are not present in the *Saccharomyces cerevisiae* homolog Eco1 (Figure 1). Regardless, MCM-Eco1 binding, albeit through a distinct mechanism, is conserved in yeast. Yeast two-hybrid and pull-down assays performed using recombinant proteins expressed in bacteria demonstrated that Eco1 indeed binds Mcm2 [84]. The Eco1-Mcm2 association in yeast requires Eco1 amino acids 63 and 70 (Figure 1). Reciprocally, two domains within Mcm2, located between amino acids 10–60 and 121–180, promote Mcm2 binding to Eco1 in yeast [84]. Unlike ESCO2, which associates with multiple MCM subunits, yeast Eco1 appears only to bind Mcm2 and through a site not present in human ESCO2 [57,78,84]. The specificity of Eco1-MCM association appears to be critical. Genetically tethering Eco1 to Mcm2, but not Mcm6, rescues *eco1* cell growth defects, suggesting that Mcm6 binding is not sufficient to promote cohesion acetylation outside the context of PCNA [67]. In combination, these results reveal that the mechanisms through which Eco1 is recruited to the DNA replication fork have evolved numerous times, or at least redundant to PCNA binding, to allow for the hand-off from pre-RC MCMs to the post-fork context of PCNA in which sister chromatids emerging from the fork can be tethered together (Figure 2).

### 4.2. Bre1 and Rtt101-Mms1-Mms22 E3-Ubiquitin Ligase Complexes Each Maintain Eco1 Position at the DNA Replication Fork

After MCM helicase-dependent recruitment to pre-RCs, the hand-off of Eco1/ESCO2 to a post-replication fork context appears to extend beyond binding to PCNA. In fact, two additional fork-associated factors promote Eco1 recruitment. The first factor, Bre1, is part of an E3 ubiquitin ligase (in association with Rad6) that modifies histone H2B and thus links cellular processes such as transcription regulation and DSB repair to DNA replication [85]. Early findings revealed that *bre1*Δ mutant cells exhibit sister chromatid cohesion defects [86]. Moreover, the absence of Bre1 results in decreased levels of chromatin-bound Eco1 as well as diminished Smc3 acetylation [75]. Bre1 promotes the association of several factors, such as Mcm10, Ctf4, and Ctf18, to the DNA replication fork [75]. The mechanism through which Bre1 impacts Eco1 recruitment, in coordination with these other factors, and how that recruitment augments that provided by PCNA and MCM helicase, requires further analysis.

A second ubiquitin ligase complex, comprising Rtt101, Mms1, and Mms22, also promotes Eco1 recruitment to the DNA replication fork. Eco1 physically interacts with Mms22 through lysine residue 61 and glycine residue 63, mutations of which result in a loss of Eco1 binding to Mms22, reduced Smc3 acetylation, and increased cohesion defects [61]. The Rtt101-Mms1-Mms22 complex appears to bind Eco1 cooperatively with PCNA in that Mms22 mutated in both Eco1 and PCNA binding results in a significant reduction in Smc3 acetylation and cell viability. In combination, these findings provide evidence that Mms22 and PCNA work in concert to promote Eco1-dependent cohesin acetylation [61].

### 4.3. Chl1 DNA Helicase

Originally identified in a yeast chromosome loss screen, numerous studies revealed that Chl1 helicase is critical for cohesion establishment and physically interacts with Eco1 [76,87,88,89]. Although *CHL1* is not an essential gene, *CHL1* deletion is synthetically lethal when combined with mutation of either *eco1* or *ctf18* [76,90]. The link between Chl1 and Eco1 highlights an exciting link to DNA repair pathways of clinical relevance. For instance, yeast Chl1 is the homolog of human DNA helicases ChlR1/DDX11 and BACH1/BRIP1/FANCJ [76]. ChlR1 (Chl1) associates with DNA fork protection complex (FPCs) subunits that include Timeless (yeast Tof1/Swi1), Tipin (yeast Csm2/Swi3), and Claspin (yeast Mrc1), as well as the GINS-Ctf4 complex, which together promote sister chromatid cohesion [91,92,93,94]. ChlR1/DDX11 mutation leads not only to cohesion defects, but also developmental maladies such as Warsaw breakage syndrome [95]. BACH1 binds the tumor suppressor BRCA1 such that BACH1 mutation leads to defects in DNA repair that contribute to numerous forms of cancer [96]. Biochemical studies revealed that Chl1 can displace proteins (that may otherwise impede replication fork progression) and also resolve secondary DNA structures that arise during DNA replication [97,98,99]. The resolution of secondary structures in the post-fork context is likely a critical aspect of cohesion establishment given that loss of Chl1 results in reduced Scc2 binding (and subsequently, cohesin binding) to chromatin [100,101,102].

## 5. New Perspectives—A Novel Model for DNA Replication-Dependent Cohesion Establishment

The field of cohesion establishment remains in a state of flux. For instance, although early studies revealed that Eco1-dependent cohesion establishment is intimately coupled to DNA replication through PCNA [33,54,55,56,57], it is now clear that Eco1/ESCO2 recruitment to the fork is mediated by numerous DNA replication factors. Moreover, Eco1 recruitments appear to have evolved in independent ways, highlighting the importance of replication-coupled cohesion establishment. Establishment models have similarly transformed over time. The founding model of replication-coupled cohesion establishment posited that cohesins are loaded onto each sister as they exit the replication fork and, in a post-DNA polymerase context that involves both PCNA and Eco1, are converted to a dimeric or clustered tethering competent state [33,56]. This active mechanism of cohesion establishment gave way to a replication-through-a-ring model. That model posited that huge cohesin rings are pre-loaded in G1, requiring only that the DNA replisome squeeze through the rings to passively establish cohesion [34,103,104,105,106]. More recent studies have largely refuted the notion of passive cohesion and instead support some mechanism in which Eco1-dependent establishment occurs in a post-fork (PCNA/MCM/E3 ligase) context in which cohesins, decorating both sisters, form dimers or clusters [33,40,41,42,43,44,45,56,67,107,108,109,110,111,112,113,114] (Figure 2). As the field continues to converge on mechanisms through which cohesin deposition and cohesion establishment occur in a post-fork context (PCNA and other DNA replication factors), new questions arise regarding how these activities are coordinated with histone deposition, chromatin-modifying enzymes, Okazaki maturation, and other forms of structural DNA machinations [115].

Cohesin and Eco1/ESCO2, of course, play roles beyond sister chromatid cohesion. For instance, mutation of either cohesins or Eco1 results in chromosome compaction defects, DNA repair defects, and transcriptional dysregulation [30,33,116,117]. These activities are genetically separable. For instance, deletion of *RAD61/WAPL* rescues the condensation defects, but not the cohesion defects, that otherwise are present in *eco1* mutant cells [62,118]. Conversely, deletion of *ELG1* rescues the cohesion defects, but not the condensation defects, that otherwise are present in *eco1* mutant cells [59,60,62]. Overexpression of Mms22, a component of the E3-ubiquitin ligase and PCNA binding partner, also rescues *eco1* mutant cell viability and cohesion defects, but the extent to which this impacts chromosome compaction defects remains untested [61].

Of immediate clinical relevance is the impact that *ESCO2* and cohesin gene mutations play in development. Cornelia de Lange syndrome/CdLS (individuals that harbor clinical mutations of cohesin or cohesin deposition gene) and Roberts syndrome/RBS (individuals that harbor clinical *ESCO2* mutation) are severe developmental maladies. CdLS and RBS phenotypes include phocomelia, organ malformation, craniofacial abnormalities, and intellectual disabilities [119,120,121,122,123,124]. It is now clear that both CdLS and RBS are transcriptional maladies, with recent evidence that these cohesinopathies are intimately linked to thalidomide teratogenicity [125,126]. Understanding the structural basis between the tenacious binding between sister chromatids that occurs during mitosis, and the dynamic tethering of DNA loci that must remain responsive to external cues to regulate transcription during G1, represents one of the remaining challenges for future research.
